# Test–Retest Reliability and Translation of the Musculoskeletal Screening Protocol Questionnaire Used in the Swedish Armed Forces

**DOI:** 10.1093/milmed/usac082

**Published:** 2022-04-01

**Authors:** Marie Kierkegaard, Matthias Tegern, Lisbet Broman, Alexandra Halvarsson, Helena Larsson

**Affiliations:** Department of Neurobiology, Care Sciences and Society, Division of Physiotherapy, Karolinska Institutet, Stockholm 141 83, Sweden; Women’s Health and Allied Health Professionals Theme, Medical Unit Occupational therapy and Physiotherapy, Karolinska University Hospital, Stockholm 141 86, Sweden; Academic Specialist Center, Center of Neurology, Stockholm Health Services, Stockholm 113 65, Sweden; Department of Neurobiology, Care Sciences and Society, Division of Physiotherapy, Karolinska Institutet, Stockholm 141 83, Sweden; Department of Community Medicine and Rehabilitation, Unit of Physiotherapy, Umeå University, Umeå 901 87, Sweden; Department of Neurobiology, Care Sciences and Society, Division of Physiotherapy, Karolinska Institutet, Stockholm 141 83, Sweden; Department of Neurobiology, Care Sciences and Society, Division of Physiotherapy, Karolinska Institutet, Stockholm 141 83, Sweden; Women’s Health and Allied Health Professionals Theme, Medical Unit Occupational therapy and Physiotherapy, Karolinska University Hospital, Stockholm 141 86, Sweden; Department of Neurobiology, Care Sciences and Society, Division of Physiotherapy, Karolinska Institutet, Stockholm 141 83, Sweden

## Abstract

**Introduction:**

Musculoskeletal disorders (MSDs) in military personnel are common, and it is important to identify those at risk so that appropriate preventive and rehabilitative strategies can be undertaken. The Musculoskeletal Screening Protocol (MSP) questionnaire is part of the implemented prevention strategy to reduce MSDs in the Swedish Armed Forces. The aims of this study were to evaluate the questionnaire’s reliability and to translate it into English.

**Materials and Methods:**

One-week test–retest reliability of the questionnaire was evaluated in a sample of 35 Swedish military personnel. Reliability was evaluated by calculations of Cohen’s kappa or quadratic-weighted kappa. Percent agreement was used as a parameter for measurement error. Translation into English included forward and backward translations and expert committee discussions.

**Results:**

Kappa values relating to physical complaints/injuries were excellent (>0.75) except for knee and lower leg MSDs and for the intensity ratings, where Kappa values were mostly interpreted as fair-to-good (0.4-0.75). Kappa values of items pertaining physical performance, physical activity and exercise, eating and tobacco habits, sleep, and perceived health ranged between 0.72 and 1. Kappa values for feeling mentally or physical prepared were 0.47 and 0.65, respectively. Most percentage agreement values ranged between 90% and 100%. The English version was found to be satisfactorily equivalent to the Swedish MSP questionnaire.

**Conclusion:**

The Swedish MSP questionnaire was found to be highly reliable and was satisfactorily translated into English. This provides support for the questionnaire’s ability to trustworthily capture the prevalence of MSDs and perceived health in military personnel. Future research is warranted on the psychometric properties of the English MSP questionnaire.

## INTRODUCTION

It is well known that musculoskeletal disorders (MSDs) in military personnel are common. Recent publications show that musculoskeletal injury rates are high,^1–6^ and besides impacting personnel health and military readiness,^1^ they also result in substantial financial burden.^5,6^ Among identified significant risk factors for MSDs are previous injuries^7,8^ and poor cardiorespiratory and muscular fitness.^7–11^ Detecting MSDs in the military population is crucial because military personnel of all categories are at risk of developing MSDs during their career. To identify those at risk early on so that appropriate preventive and rehabilitative strategies can be undertaken is therefore of utmost importance. Research shows that timely identification, early management of MSDs, and leadership awareness of injuries and injury prevention are important contributors to the effectiveness of injury prevention strategies.^12–14^ Commonly used methods in identifying MSDs and injury risk include self-report questionnaires and various screening tests.^15–21^ Important aspects of such methods are that they show sound psychometric properties, e.g., that they are reliable.

The Musculoskeletal Screening Protocol (MSP), comprising a questionnaire and physical tests, was originally developed for the Swedish Air Force and the Swedish Parachute Association with the main purpose of identifying pilots and parachutists with MSDs. It was later extended to also include military conscripts at risk of premature discharge from the Swedish Armed Forces (SwAF).^22,23^ The self-administrated MSP questionnaire has since then been used to investigate the 1-year and point prevalence of MSDs as well as perceived health both before and after military training as well as before and after deployment.^24–28^ The MSP outcome sorts the personnel to different intervention categories such as primary and secondary prevention or rehabilitation, as described and regulated in the SwAF guidelines/handbook “Optimize training and exercise.”^29^ The MSP is used both in practice and in previous and on-going research (around 14,000 questionnaires have been collected since the year 2000), including cross-sectional, longitudinal, and experimental studies.^24–28^ The reliability of the MSP questionnaire was explored in 28 conscripts, answering the questionnaire 14 days apart, as part of a thesis work in 2009.^22^ The findings were not presented in detail in the thesis, and the results were only summarized as ranging from poor to excellent reliability.^22^

The MSP questionnaire has over time been slightly revised and now consists of one version used before military training/deployment and another extended version for follow-up after military training/deployment. Both include questions regarding MSDs in 10 anatomical parts (neck, upper back, lower back, shoulder, elbow, hand, hip, knee, lower leg, and foot), physical performance, physical activity and exercise, eating and tobacco habits, sleep, motivation and mental/physical preparation, and perceived health. The follow-up questionnaire includes additional questions on physical exposure during the military training/deployment and perceived change in physical fitness. The MSP questionnaire is part of the implemented prevention strategy to reduce MSDs in the SwAF. In order to be able to share the questionnaire internationally, a translation to English was needed. Furthermore, a test–retest reliability study was considered relevant because the questionnaire had gone through some changes. Thus, the aims of this study were to evaluate the test–retest reliability of the MSP questionnaire used before military training/deployment and to translate the questionnaire into English.

## METHODS

### Procedure Reliability

A test–retest design was applied to evaluate the reliability of the Swedish MSP questionnaire used before military training/deployment. A convenience sample of military personnel in the SwAF were recruited as participants. Inclusion criteria were native Swedish adults >18 years of age and having experience of field work. After informed consent, participants filled out the first MSP questionnaire, which was then put in a pre-paid envelope and sent to the first author (MK). Participants were then given the second MSP questionnaire and another pre-paid envelope. They were informed to fill out this second MSP questionnaire a week later and to return it by post to the first author (MK). The time interval of 1 week was considered appropriate for participants to remain stable and not remember their previous answers. The regional board of ethics in Stockholm granted approval for the study (Dnr 2015/493-32), and procedures were conducted in accordance with the Declaration of Helsinki.

### Measure

The first part of the MSP questionnaire on physical complaints/injuries includes dichotomized questions (yes or no answers) on MSDs during the last year and at present in 10 anatomical parts and whether the MSDs have affected the ability to work. Those who are presently suffering from MSDs rate the intensity of their complaints/injuries on a 11-point numeric rating scale (NRS) ranging from 0 (not at all) to 10 (worst imaginable) as well as the frequency (rarely, frequently, or daily) of these complaints/injuries. There are dichotomized questions exploring if the respondent has been off duty during the last 12 months and whether he/she has been seeking care for the complaints. The second part of the MSP questionnaire addresses physical performance, i.e., how the physical part of previous work has been managed regarding muscular strength and cardiorespiratory fitness, both rated on a 5-point ordinal scale from very poorly to very well. The next part concerns physical activity and exercise. Frequency of light-intensity and moderate-to-vigorous physical activity and exercise, respectively, is rated on a 6-point ordinal scale from never to 4 times/week or more. Respondents also report what kind of physical exercise they perform and how often. The part on eating and tobacco habits includes questions (yes or no answers) exploring if respondents eat breakfast every day, eat a cooked meal twice a day, or use snuff or smoke. Perceived sleep problems are rated on a 6-point ordinal scale from never to all the time. Questions on whether respondents are motivated to undergo military training/deployment and if they feel sufficiently mentally and physically prepared, respectively, are answered with yes or no. The last part of the MSP questionnaire includes five questions on perceived health, i.e., how they experience their physical and mental health, physical and social environment, and work ability, all rated on a 7-point NRS from 1 (very poor) to 7 (excellent).

### Translation Procedure

The translation procedure began with translations from Swedish to English performed separately by one independent professional translator (British English) and two bilingual non-professional individuals familiar with terminology. These translations were then discussed by an expert committee consisting of a research assistant and three physiotherapists with both clinical and research experience within the field in round table discussions until consensus was reached. This resulted in a first English version that was sent out to two bilingual military personnel. This resulted in minor comments on wording. The expert committee then met to discuss these comments and to reach a satisfactory English version. Back translations were then performed separately by one independent professional translator and one bilingual non-professional individual (not previously involved in the study). The expert committee reviewed the back translations against the original source to ensure the conceptual equivalence of the translations. Because no conceptual discrepancies were found in wording, the expert committee agreed on a final English version of the MSP questionnaire. There was no quantitative analysis performed in the process for interpretation of the translations.

### Statistical Analyses

Descriptive statistics were used to present the data, i.e., mean, standard deviation (SD), median, interquartile range, minimum and maximum value, number, and percentage. The test–retest reliability of the MSP questionnaire was evaluated by analyses of the measurement reliability and measurement error.^30,31^ Reliability analyses included calculations of Cohen’s kappa (K) values or quadratic-weighted kappa (Kw) values when questions were answered on ≥6-point ordinal scales.^30^ Kappa coefficients were based on listwise deletion of missing data as recommended.^32^ The 95% CI was calculated for both K and Kw (i.e., K/Kw ± 1.96 × standard error). K and Kw values greater than 0.75 were interpreted as excellent, values between 0.40 and 0.75 were considered fair-to-good agreement, and values below 0.40 were considered as poor agreement.^33^ Percent agreement (PA) was used as a parameter for measurement error.^30^

## RESULTS

### Reliability

Thirty-five military personnel (14 men, 21 women) agreed to participate. Their mean (SD) age was 45 (11) years ranging from 24 to 63 years. Their mean (SD) weight was 74.2 (10.6) kg ranging from 54 to 97 kg, and their mean (SD) body mass index was 24.4 (2.2) kg/m^2^ ranging from 19.8 to 30.1 kg/m^2^. No other personal data were collected. The mean time between test and retest was 8 days, ranging from 4 to 15 days.

Results of the MSP questionnaire’s first part on physical complaints/injuries are presented in Tables I-III. K values were in general excellent, i.e., >0.75, except for knee and lower leg MSDs and for intensity ratings where K values were mostly interpreted as fair-to-good. Approximately a third of the participants reported lower back problems during the last year. Various lower extremity complaints/injuries were most common at present. Few, however, perceived that these complaints/injuries had affected their ability to work.

**TABLE I.** Reported Prevalence of Musculoskeletal Disorders Last Year and at Present and Test–Retest Reliability Results (*n* = 35)

Physical complaints/injuries				
Last year	Yes, test/retest, *n*	Kappa	95% CI	PA
Neck	4/4	1.00	1.00-1.00	100
Upper back	5/4 ^a^	0.87	0.63-1.12	97
Lower back	11/11	1.00	1.00-1.00	100
Shoulder, left	5/5	1.00	1.00-1.00	100
Shoulder, right	8/8	1.00	1.00-1.00	100
Elbow	2/2	1.00	1.00-1.00	100
Hand	0/0	#	#	100
Hip/buttock	4/4	1.00	1.00-1.00	100
Knee. left	7/7 ^b^	0.82	0.58-1.06	97
Knee, right	7/6 ^a^	0.91	0.72-1.09	97
Lower leg	6/4 ^b^	0.77	0.46-1.07	94
Foot	6/6	1.00	1.00-1.00	100
At present	Yes, test/retest, *n*	Kappa	95% CI	PA
Neck	2/3 ^a^	0.79	0.38-1.19	97
Upper back	0/0	#	#	100
Lower back	4/4	1.00	1.00-1.00	100
Shoulder, left	4/4	1.00	1.00-1.00	100
Shoulder, right	4/3 ^a^	0.84	0.54-1.14	97
Elbow	1/1	1.00	1.00-1.00	100
Hand	0/0	#	#	100
Hip/buttock	4/4	1.00	1.00-1.00	100
Knee, left	6/4^b^	0.77	0.46-1.07	94
Knee, right	2/1^a^	0.65	0.03-1.28	97
Lower leg	5/2^c^	0.53	0.09-0.98	91
Foot	5/4^a^	0.87	0.63-1.12	97

Abbreviations: CI: confidence interval, PA: percentage agreement, # Kappa could not be calculated due to constant variables.

a One participant changed category.

b Two participants changed category.

c Three participants changed category.

**TABLE II.** Number of Participants Reporting That Their Present Musculoskeletal Disorders Had Affected Their Ability to Work and Test–Retest Reliability Results (*n* = 35)

	Yes, to some extent, test/retest, *n*	Yes, to a great extent, test/retest, *n*	Kappa	95% CI	PA
Neck	0/0	0/0	#	#	100
Upper back	1/1	0/0	1.00	1.00-1.00	100
Lower back	2/2	0/0	1.00	1.00-1.00	100
Shoulder, left	1/1	0/0	1.00	1.00-1.00	100
Shoulder, right	2/2	0/0	1.00	1.00-1.00	100
Elbow	0/0	0/0	#	#	100
Hand	0/0	0/0	#	#	100
Hip/buttock	0/0	1/1	1.00	1.00-1.00	100
Knee, left	2/1 ^a^	0/0	0.65	0.03-1.28	97
Knee, right	2/0	0/0	#	#	94
Lower leg	1/1	0/0	1.00	1.00-1.00	100
Foot	2/2	1/0^a^	0.79	0.39-1.19	97

Abbreviations: CI: confidence interval, PA: percentage agreement, # Kappa could not be calculated due to constant variables.

a One participant changed category.

**TABLE III.** Ratings of Intensity on an NRS Ranging From 0 (Not at All) to 10 (Worst Imaginable) and Frequency of Musculoskeletal Disorders at Present and the Test–Retest Reliability Results (*n* Indicates Number of Reported Complaints/Injuries)

		Test	Retest			
Intensity (NRS)	*n*	Median (IQR) min–max	Median (IQR) min-max	Weighted Kappa	95% CI	PA
All	28	3 (2-5) 2-8	3 (2-4) 2-8	0.62	0.35-0.90	46
Neck/back	6	2 (2-4) 2-5	3 (2-5) 2-7	0.52	−0.09-1.13	33
Upper extremity	8	3 (2-4) 2-5	2.5 (2-3,5) 2-4	0.60	0.29-0.92	50
Lower extremity	14	4.5 (3-6) 2-8	3.5 (3-6) 2-8	0.58	0.18-0.99	50
		Test	Retest			
Frequency	*n*	Rarely/frequently/daily, *n*	Rarely/frequently/daily, *n*	Kappa	95% CI	PA
All	23	3/11/9	3/12/8	0.78	0.56-1.01	87
Neck/back	6	1/3/2	0/4/2	0.70	0.23-1.17	83
Upper extremity	6	1/4/1	2/3/1	0.71	0.21-1.21	83
Lower extremity	11	1/4/6	1/5/5	0.84	0.55-1.14	91

Abbreviations: CI: confidence interval, IQR: interquartile range, min: minimum, max: maximum, NRS: Numeric rating scale, PA: percentage agreement.

In all, 19 participants reported in total 32 complaints/injuries at present (8 participants in 1 anatomical part, 9 participants in 2 anatomical parts, and 2 participants in 3 anatomical parts). There were missing data from four and nine participants in intensity and frequency ratings, respectively. Thus, descriptive and reliability results of intensity ratings are reported for 28 present MSDs (Table III). Median intensity ratings were in general at the lower end, except for lower extremity complaints/injuries. As for reliability, 13 of the 28 intensity ratings were consistent, whereas 15 changed one or more categories. The frequency of the present complaints/injuries was mostly rated as frequently, i.e., a few times/week. None had been off duty due to the complaints/injuries. Nine of the 19 participants who had complaints/injuries at present reported that they had been seeking care for these complaints at the time of testing, and eight at the time of retesting (K = 0.68; 95% CI, 0.35-1.01; PA = 84.)

How participants perceived that they managed the physical part of their work regarding muscular strength was rated as very well by 20 participants and quite well by 9 participants at both test and retest, while 3 participants changed category and 3 had missing data (K = 0.79; 95% CI, 0.56-1.01; PA = 91). Ratings on managing the physical part of their work regarding cardiorespiratory fitness were consistently rated as very well by 19 participants, as quite well by 7 participants, and as adequate by 2 participants, while 4 participants changed category and 3 participants had missing data (K = 0.74; 95% CI, 0.50-0.98; PA = 88).

Ratings of light-intensity physical activity and exercise were consistent for 31 participants, while 3 participants changed category and 1 participant had missing data (Kw = 0.90; 95% CI, 0.75-1.05; PA = 91). Approximately two-thirds reported a frequency of light-intensity physical activity of ≥4 times/week. Ratings of moderate-to-vigorous physical activity and exercise were consistent for 34 participants (Kw = 0.98; 95% CI, 0.94-1.02; PA = 97), where a third each reported moderate-to-vigorous physical activity 2 times/week, 3 times/week, and ≥4 times/week. All participants performed some sort of physical exercise, and all but one reported the weekly frequency. The median frequency was 2 times/week for both muscular strength and cardiorespiratory fitness exercise, and 3 times/week for those performing a combination of strength and cardiorespiratory exercise (Table IV). K values varied from 0.72 to 0.88, indicating good to excellent agreement beyond chance (Table IV).

**TABLE IV.** Reported Frequency (Times/Week) of Physical Exercise and Ratings of Perceived Health on an NRS Ranging from 1 (Very Poor) to 7 (Excellent) and the Test–Retest Reliability Results

		Test	Retest			
Physical exercise (times/week)	n	Median (IQR) min–max	Median (IQR) min–max	Kappa	95% CI	PA
Strength	12	2 (1-2.5) 1-3	2 (1.3-2.5) 1-3	0.88	0.66-1.10	92
Cardiorespiratory	17	2 (1.5-3) 1-7	2 (2-3) 1-7	0.77	0.55-0.99	82
Combination	17	3 (2-4.5) 1-6	3 (2-4.5) 1.5-6	0.72	0.50-0.95	76
		Test	Retest			
Perceived health (NRS)	*n*	Median (IQR) min–max	Median (IQR) min–max	Weighted Kappa	95% CI	PA
Physical state	34	6 (5-6) 3-7	6 (5-6) 4-7	0.80	0.68-0.91	71
Mental state	35	6 (5-7) 4-7	6 (5.5-6) 4-7	0.87	0.77-0.97	83
Physical environment	35	6 (5-6) 4-7	6 (5-6) 4-7	0.81	0.64-0.98	80
Social environment	35	6 (5-6) 2-7	6 (5-6) 3-7	0.82	0.71-0.93	71
Work ability	35	6 (6-7) 5-7	6 (6-7) 5-7	0.84	0.72-0.96	83

Abbreviations: CI: confidence interval, IQR: interquartile range, min: minimum, max: maximum, NRS: Numeric rating scale, PA: percentage agreement.

All but three participants reported eating breakfast at both test and retest (K = 1.00; 95% CI, 1.00-1.00; PA = 100). Thirty-one participants consistently reported eating a cooked meal twice a day, three did not, and one changed category (K = 0.84; 95% CI, 0.54-1.14; PA = 97). Six of the 35 participants reported using snuff at both test and retest (K = 1; 95% CI, 1.00-1.00; PA = 100), and the number of portions/day varied from 3 to 20 (K = 0.81; 95% CI, 0.48-1.13; PA = 83). Two participants were smokers and reported smoking 7 and 15 cigarettes/day, respectively (K = 1.00; 95% CI, 1.00-1.00; PA = 100). Participants’ answers on perceived sleep problems were consistent, and 2 participants had them frequently, 9 had them sometimes, 17 had them rarely, and 7 reported that they never had sleep problems (Kw = 1.00; 95% CI, 1.00-1.00; PA = 100).

There were missing data from six participants concerning questions on motivation and mental/physical preparation. Thirty participants stated that they were motivated to undergo military training/deployment at test and 29 at retest (K could not be calculated due to constant variables). At test, 27 participants reported feeling sufficiently mentally prepared to undergo military training/deployment and 3 participants did not, whereas at retest all but one felt sufficiently prepared (K = 0.47; 95% CI, −0.13 to 1.07; PA = 93). As for feeling sufficiently physically prepared, 27 consistently reported yes and one reported no, while one participant changed category (K = 0.65; 95% CI , 0.02-1.28; PA = 97).

Participants’ ratings on perceived health were in general in the range from good to excellent (Table IV). A few did, however, rate the condition of their social environment as poor. The Kw values ranged from 0.80 to 0.87, indicating excellent reliability (Table IV).

### Translation

The English version of the MSP questionnaire for use before military training/deployment was found to be satisfactorily equivalent to the Swedish questionnaire. During the translation process, some adjustments in wording concerning gradings were made for linguistic clarity. The final English version is presented in a supplementary file.

## DISCUSSION

The aims of the present study were to evaluate the test–retest reliability of the MSP questionnaire and to translate the questionnaire into English. The results indicate that the test–retest reliability and the English translation were satisfactory.

Descriptive data from the MSP questionnaire showed that MSDs were most common in the low back, knee, lower leg, and foot. These data are in line with international reports^3,6,17,34^ and corroborate previous findings of the 1-year and point prevalence of MSDs in male conscripts,^23^ military personnel,^25^ and male deployed soldiers^28^ in the SwAF. In accordance with previous research,^28^ the median intensity ratings were at the lower end. In addition, reported physical activity levels and perceived health ratings in the present study are like previous studies in Swedish soldiers.^24,25,28^ Taken together, these data imply that the study sample can be considered representative of military personnel in both the SwAF and international military cohorts.

Test–retest reliability was evaluated by analyses of the measurement reliability and measurement error as proposed by the international initiative COnsensus-based Standards for the selection of health Measurement INstruments (COSMIN).^30,31^ The measurement reliability is defined according to COSMIN as “the proportion of the total variance in the measurements which is because of true differences among patients,”^31^ and it gives information on how well patients can be distinguished from each other despite measurement error. Measurement error is defined by COSMIN as “the systematic and random error of a patient’s score that is not attributed to true changes in the construct to be measured”^31^ and gives information on how close scores are for repeated measurements. Because variables in the MSP questionnaire were all categorical, i.e., nominal or ordinal, the parameters of reliability were K and Kw. Because data on the categorical level only classify or order, the measurement error cannot be expressed in units of the measurement. It can, however, be evaluated by calculations of percentage of agreement, i.e., the percentage of measurements that are classified in the same categories.

Overall, our results show that the MSP questionnaire was reliable with acceptable measurement errors. Of the 66 calculated K or Kw values, 44 (67%) were considered excellent, 14 (21%) were in the range of fair to good, and 8 (12%) could not be calculated due to constant variables. As for measurement errors, most PA values were in the range of 90-100%, and only a few were below 80%. The only variables that showed both low Kw and PA values were intensity ratings of present MSDs. Although an NRS is considered appropriate for assessment of pain,^35,36^ issues referring to inconsistent findings for test–retest reliability and responsiveness have been raised.^37^ Furthermore, qualitative research has shown that the timeframe might be particularly problematic, i.e., an NRS assessing pain at the moment is biased by symptom fluctuations.^38^ Thus, our findings of low Kw and PA values can be explained by daily fluctuations in perceived intensity and perhaps also to varied physical exposure.

Although it is well recognized that MSDs are a significant challenge to military readiness, the literature on screening tools for preventive and rehabilitative purposes in a military context is scarce. In addition, the evaluation of psychometric properties like reliability is almost non-existent. We could only identify four other studies^15,18,21,39^ that explored reliability. Of these, two studies^15,18^ focused on test–retest reliability of questionnaires. However, Coppack et al.^15^ calculated intraclass correlation coefficients even though their data were ordinal and thus used an incorrect method. In the study by Robinson et al.,^18^ K or Kw were used to assess the 2-week test–retest reliability for categorical items pertaining to the domains for physical activity, injury history, diet, alcohol, and smoking included in the Military Pre-training Questionnaire. Their K values ranged from 0.45 to 0.86, and their Kw values ranged from 0.11 to 0.91, where the lowest values were found for diet items. Like in our study, items relating to physical activity, injuries/complaints, and smoking were all found to demonstrate substantial to almost perfect reliability.

The translation process was in line with published guidelines,^40^ although with some deviations. For example, the synthesis of the forward translations was made by the expert committee and not the translators and a recording observer as proposed by Beaton et al.^40^ Nor has a pre-test of the translated questionnaire in a target population been performed because this cannot be performed in Sweden. Nevertheless, we did follow the other stages such as having at least two independent forward and backward translations and an expert committee to achieve cross-cultural equivalence. Further research is, however, warranted to explore reliability and other aspects of validity of the English MSP questionnaire.

This study has its strengths and weaknesses that must be considered when interpreting the results. A limitation is the sample size where around 50 participants have been recommended for reliability studies.^30^ Further, we have limited information on participants’ background data because anonymity was considered important. However, the similarity in descriptive results to previous studies gives evidence of the sample’s representativity of military personnel. In the light of the limited studies on psychometric properties on screening tools for use in military populations, our study contributes to new and valuable information. We have shown that the MSP questionnaire used before military training/deployment is reliable.

## CONCLUSIONS

In conclusion, the Swedish MSP questionnaire was found to be highly reliable and was satisfactorily translated into English. This provides support for the questionnaire’s ability to trustworthily capture the prevalence of MSDs and perceived health in military personnel. Future research is warranted on the psychometric properties of the English MSP questionnaire.

## Supplementary Material

usac082_Supp

## Data Availability

The datasets used and/or analyzed during the current study are available from the corresponding author on reasonable request.
