# Supplementary material for: Test–Retest Reliability and Translation of the Musculoskeletal Screening Protocol Questionnaire Used in the Swedish Armed Forces
Source: Mil Med. 2022 Apr 1;188(7-8):2318–24. doi: 10.1093/milmed/usac082 (PMC10362998; doi:10.1093/milmed/usac082)
Supplement: usac082_Supp [file usac082_supp.zip › usac082_Supp/MSP questionnaire.pdf]

Questionnaire

Before military training/deployment

Date: ....

Name: ....

Date of birth: ....

Male ☐ Female ☐ Body height: .... Body weight: ....

Unit: ....

Position no: .... Position: ....

What has been your main occupation before reporting for duty?

Studies ☐ Which? ....

Work ☐ Which? ....

Other ☐ Describe ....

Email and/or mobile phone (to be contacted on): ....

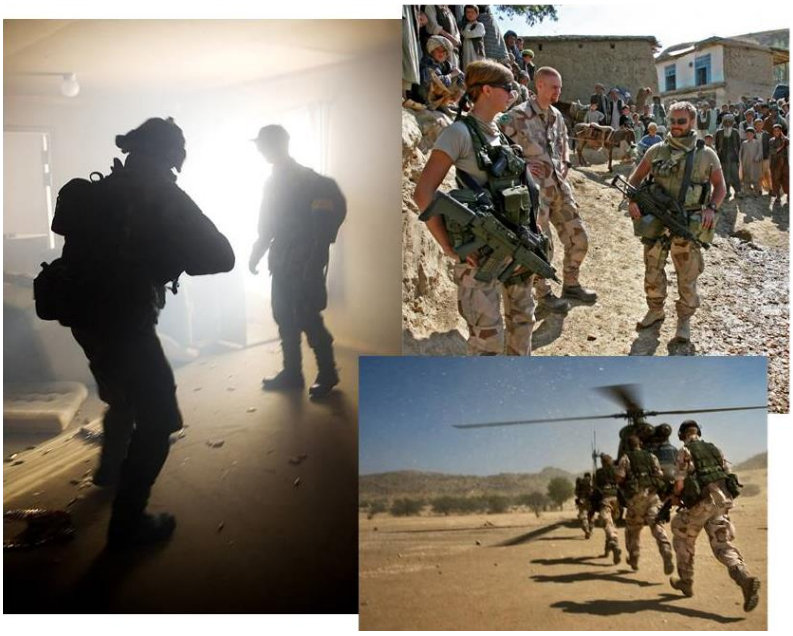

Photo: Combat Camera, Swedish Armed Forces

Physical complaints/injuries

1. Have you experienced any physical complaints or injures in any part of your body?

	Complaints/ injuries over the **past 12 months**		Complaints/ injuries **at present**?		Have these complaints/injuries affected your ability to work?		
Neck	☐ No	☐ Yes	☐ No	☐ Yes	☐ No	☐ Yes, to some extent	☐ Yes, to a great extent
Upper back	☐ No	☐ Yes	☐ No	☐ Yes	☐ No	☐ Yes, to some extent	☐ Yes, to a great extent
Lower back	☐ No	☐ Yes	☐ No	☐ Yes	☐ No	☐ Yes, to some extent	☐ Yes, to a great extent
Pelvis/hip	☐ No	☐ Yes	☐ No	☐ Yes	☐ No	☐ Yes, to some extent	☐ Yes, to a great extent
Shoulder, left	☐ No	☐ Yes	☐ No	☐ Yes	☐ No	☐ Yes, to some extent	☐ Yes, to a great extent
Shoulder, right	☐ No	☐ Yes	☐ No	☐ Yes	☐ No	☐ Yes, to some extent	☐ Yes, to a great extent
Elbow	☐ No	☐ Yes	☐ No	☐ Yes	☐ No	☐ Yes, to some extent	☐ Yes, to a great extent
Hand	☐ No	☐ Yes	☐ No	☐ Yes	☐ No	☐ Yes, to some extent	☐ Yes, to a great extent
Knee, left	☐ No	☐ Yes	☐ No	☐ Yes	☐ No	☐ Yes, to some extent	☐ Yes, to a great extent
Knee, right	☐ No	☐ Yes	☐ No	☐ Yes	☐ No	☐ Yes, to some extent	☐ Yes, to a great extent
Lower leg	☐ No	☐ Yes	☐ No	☐ Yes	☐ No	☐ Yes, to some extent	☐ Yes, to a great extent
Foot	☐ No	☐ Yes	☐ No	☐ Yes	☐ No	☐ Yes, to some extent	☐ Yes, to a great extent

2. **If you stated that you are suffering from complaints/injuries at present**, specify on the line the parts of your body affected by ***complaints now***. Tick in the boxes to indicate the intensity and frequency, respectively, of your complaints.

How **frequently** have you had these complaints?

2a. ....

	0	1	2	3	4	5	6	7	8	9	10		
Not at all	☐	☐	☐	☐	☐	☐	☐	☐	☐	☐	☐	Worst imaginable	☐ *Rarely*- a few times/month<br>☐ *Frequently*- a few times/week<br>☐ *Daily*- more or less

2b. ....

	0	1	2	3	4	5	6	7	8	9	10		
Not at all	☐	☐	☐	☐	☐	☐	☐	☐	☐	☐	☐	Worst imaginable	☐ *Rarely*- a few times/month<br>☐ *Frequently*- a few times/week<br>☐ *Daily*- more or less

2c. ....

	0	1	2	3	4	5	6	7	8	9	10		
Not at all	☐	☐	☐	☐	☐	☐	☐	☐	☐	☐	☐	Worst imaginable	☐ *Rarely*- a few times/month<br>☐ *Frequently*- a few times/week<br>☐ *Daily*- more or less

2d. ....

	0	1	2	3	4	5	6	7	8	9	10		
Not at all	☐	☐	☐	☐	☐	☐	☐	☐	☐	☐	☐	Worst imaginable	☐ *Rarely*- a few times/month<br>☐ *Frequently*- a few times/week<br>☐ *Daily*- more or less

3. During the last 12 months, have you at any occasion/occasions been off duty due to these complaints/injuries? ☐ No ☐ Yes

4. Have you sought care for these complaints? ☐ No ☐ Yes

If yes,describe? ....

....

....

Physical performance

5. You who previously worked, how did you manage the *physical* part of your work/service?

With regard to muscle strength		With regard to cardiorespiratory fitness	
	☐ Very well		☐ Very well
	☐ Well		☐ Well
	☐ Adequately		☐ Adequately
	☐ Poorly		☐ Poorly
	☐ Very poorly		☐ Very poorly

Physical activity and exercise

6. Mark with a cross how often you engage in light-intensity and moderate-to-vigorous physical activity and exercise.

How often do you engage in **light-intensity physical activity and exercise** (eg. calm walks and bike rides)?		How often do you engage in **moderate-to-vigorous physical activity and exercise** (your pulse and breathing increases and you sweat)?	
	☐ Never		☐ Never
	☐ Irregular		☐ Irregular
	☐ 1 time/week		☐ 1 time/week
	☐ 2 times/week		☐ 2 times/week
	☐ 3 times/week		☐ 3 times/week
	☐ 4 times/week or more		☐ 4 times/week or more

7. What kind of physical exercise do you perform?

☐ Do not perform exercise
☐ Muscle strength .... times/week
☐ Cardiorespiratory fitness .... times/week
☐ Combination of both .... times/week
☐ Other .... times/week

Eating and tobacco habits

8. Do you eat breakfast every day? ☐ No ☐ Yes
9. Do you eat a cooked meal twice a day? ☐ No ☐ Yes
10. Do you take Scandinavian snuff (snus)? ☐ No ☐ Yes, number of portions/day? ....
11 Do you smoke? ☐ No ☐ Yes, number of cigarettes/day? ....

Sleep

12. Do you perceive that you have sleep problems?

Never	Rarely	Sometimes	Frequently	Most of the time	All the time
	(Occasionally, sometimes/year)	*(Several times/month)*	*(1-2 times/week)*	*(3-4 times/week)*	*(5 or more times/week)*
☐	☐	☐	☐	☐	☐

Motivation and mental / physical preparation

13. Are you motivated to undergo your military training/deployment? ☐ No ☐ Yes
14. Do you feel that you are sufficiently *mentally* prepared? ☐ No ☐ Yes
15. Do you feel you are sufficiently *physically* prepared? ☐ No ☐ Yes

Perceived health

The most common way to analyse your perception of your own health is to try and work out how your body and head feel compared with previously. There are no precise answers when it comes to health! Like everything else, health is a subjective experience and never precise.

How do you experience your physical health?

Here, indicate your overall perception of the state of your physical body, i.e. where you are along the line *not ill ---- ill*.

Very poor	☐	1
Poor	☐	2
	☐	3
Neither good nor poor	☐	4
Good	☐	5
	☐	6
Excellent, could not be better	☐	7

How do you experience your mental health?

Here, indicate your overall perception of your mental state, i.e. where you are along the line *feel fine ---- feel poorly*.

Very poor	☐	1
Poor	☐	2
	☐	3
Neither good nor poor	☐	4
Good	☐	5
	☐	6
Excellent, could not be better	☐	7

How do you experience the condition of your physical environment?

Here, indicate your overall perception of the condition of your physical surroundings.

Very poor	☐	1
Poor	☐	2
	☐	3
Neither good nor poor	☐	4
Good	☐	5
	☐	6
Excellent, could not be better	☐	7

How do you experience the condition of your social environment?

Here, indicate your overall perception of the condition of your social environment.

Very poor	☐	1
Poor	☐	2
	☐	3
Neither good nor poor	☐	4
Good	☐	5
	☐	6
Excellent, could not be better	☐	7

How do you experience your work ability?

Here, indicate your overall perception of your ability to manage the tasks that lie ahead.

Very poor	☐	1
Poor	☐	2
	☐	3
Neither good nor poor	☐	4
Good	☐	5
	☐	6
Excellent, could not be better	☐	7

©HLar2021
